# Physical activity influences adherence to pharmacological treatments in patients with severe mental disorders: results from the multicentric, randomized controlled LIFESTYLE trial

**DOI:** 10.3389/fphar.2023.1285383

**Published:** 2023-12-11

**Authors:** Gaia Sampogna, Mario Luciano, Matteo Di Vincenzo, Claudia Toni, Enrico D’Ambrosio, Antonio Rampino, Alessandro Rossi, Rodolfo Rossi, Mario Amore, Pietro Calcagno, Alberto Siracusano, Cinzia Niolu, Liliana Dell’Osso, Barbara Carpita, LIFESTYLE Working Group, Andrea Fiorillo

**Affiliations:** ^1^ Department of Psychiatry, University of Campania “L. Vanvitelli”, Naples, Italy; ^2^ Department of Translational Biomedicine and Neuroscience, University of Bari Aldo Moro, Bari, Italy; ^3^ Department of Biotechnological and Applied Clinical Sciences, University of L’Aquila, L’Aquila, Italy; ^4^ Department of Systems Medicine, University of Rome Tor Vergata, Rome, Italy; ^5^ Department of Clinical and Experimental Medicine, University of Pisa, Pisa, Italy

**Keywords:** adherence, physical activity, severe mental disorder, lifestyle, personalization

## Abstract

**Introduction:** Poor adherence to pharmacological treatment is frequent in people with severe mental disorders and it often causes lack of effectiveness of many psychotropic drugs. Thus, efforts should be made to improve adherence to pharmacological treatments in patients with these disorders.

**Methods:** In this paper, based on the LIFESTYLE randomized, controlled multicentric trial, we aim to: 1) assess the level of adherence in a real-world sample of patients with severe mental disorders; 2) evaluate differences in treatment adherence according to patients’ socio-demographic and clinical characteristics; 3) evaluate the impact of an innovative psychosocial intervention, on patients’ adherence to treatments. The Lifestyle Psychosocial Group Intervention consists of group sessions, focused on different lifestyle behaviours, including healthy diet; physical activity; smoking habits; medication adherence; risky behaviours; and regular circadian rhythms. At end of each session a 20-min moderate physical activity is performed by the whole group.

**Results:** The sample consists of 402 patients, mainly female (57.1%, N = 229), with a mean age of 45.6 years (±11.8). Less than 40% of patients reported a good adherence to pharmacological treatments. Adherence to treatments was not influenced by gender, age, diagnosis and duration of illness. At the end of the intervention, patients receiving the experimental intervention reported a significant improvement in the levels of adherence to treatments (T0: 35.8% vs. T3: 47.6%, *p* < 0.005). Patients practicing moderate physical activity reported a two-point improvement in the levels of adherence [odds ratio (OR): 1,542; 95% confidence intervals (CI): 1,157–2,055; *p* < 0.001], even after controlling for several confounding factors.

**Discussion:** The experimental lifestyle intervention, which can be easily implemented in the routine clinical practice of mental health centres, was effective in improving adherence to pharmacological treatments.

## Background

Adherence to treatment or medication compliance, defined as intake of medications according to the prescribed dosage provided by referring clinician and with persistence over time (World Health Organization, 2003). A different concept is that of medication persistence, referring to the act of continuing the treatment for the prescribed duration (Cramer et al, 2008). Medication adherence is fundamental to prevent relapses, improve long-term clinical and functional outcome, and reduce healthcare costs in people suffering from chronic physical or mental disorders (Davidson and Tondora, 2022; McIntyre et al., 2022). People suffering from severe mental disorders often report non-adherence to prescribed medications, ranging from 28%–52% in people suffering from major depression to 70% in patients with schizophrenia (Baylé et al., 2015). About 40% of patients stop taking their medication within a year, and up to 75% up to 2 years (Semahegn et al., 2020). Poor adherence to pharmacological treatment is also common in people suffering from other chronic diseases, such as diabetes, cardiovascular diseases or chronic obstructive pulmonary disease (COPD) (Vetrano et al., 2017; Marrie and Bernstein et al., 2021), with approximately 50% of patients not taking properly the drugs prescribed for long-term therapies (Brown and Bussell, 2011; Kim et al., 2021; Fusar-Poli et al., 2022; Mirhaj Mohammadabadi et al., 2022; Ostuzzi et al., 2022).

Lack of adherence is one of the causes for low efficacy of many pharmacological treatments and should be carefully evaluated in clinical practice. In fact, the WHO has defined an “invisible epidemic” the poor or lack of adherence to treatments, which should be tackled with any possible effective initiative (WHO, 2003).

Nonadherence to pharmacological and non-pharmacological interventions is considered a multifactorial phenomenon, including causes related to the patient, the healthcare system and the clinician (Lam and Fresco, 2015; Caqueo-Urízar et al., 2021). Patients’ adherence to medications is significantly reduced by lack of insight (Novick et al., 2015), negative beliefs about the efficacy of the medications, concerns about side effects, costs of medications, low educational level, and belonging to ethnic minority (Lemay et al., 2018). In particular, several studies have highlighted that lower insight is associated with lower adherence and a worse therapeutic relationship (Novick et al., 2015; Elowe et al., 2022; Okobi et al., 2022). Healthcare system factors mainly include polypharmacy (particularly in older adults) and care fragmentation provided by different healthcare professionals (Aggarwal et al., 2020). Clinician-related factors include excessive workload, lack of time for patient’s education about treatments, poor adoption of the shared decision-making approach (Dell’Osso et al., 2020; Caqueo-Urízar et al., 2021). Because these factors usually interact and potentiate each other, multilevel and integrated strategies are required to efficiently address poor adherence to medications.

Available interventions for improving treatment adherence have been grouped into four categories: educational, behavioural, cognitive-behavioural, and multicomponent approaches (Torres-Robles et al., 2018; Cuijpers et al., 2021; Leichsenring et al., 2022). The most frequently adopted interventions are psychoeducation (Killaspy et al., 2022; Harmancı and Yıldız, 2023), problem-solving strategies (Chatoo and Lee, 2022), and programmes aiming to promote the adoption of a shared decision-making clinical style (Fiorillo et al., 2020; Fulford and Handa, 2021; Roe et al., 2022). Despite this, adherence rates to treatments remain incredibly low, highlighting the need to develop and implement innovative and effective strategies. One of these innovative strategies is represented by the promotion of healthy lifestyle behaviours, including regular physical activity. A recent study carried out in a cardiology unit involving patients suffering from hypertension has shown the positive effect of regular physical activity on adherence to medications (Fragoulis et al., 2023) after a behavioral activation intervention. Thus, the authors concluded that there is the need for innovative research in this field for further confirmation of the positive relationship between treatments’ adherence and physical activity. Indeed, physical activity defined as any planned, systematic, and repetitive physical exercise that enhances athletic performance by improving body composition, fitness, and motor abilities (Mahindru et al., 2023), is considered a complementary treatment modality in the management and control of non-communicable diseases, including severe mental disorders, and is associated with the reduction of cardiovascular risk, morbidity and mortality (Theofilou and Saborit, 2013; Saqib et al., 2020; Arango et al., 2021; Baron and Noordsy, 2021; Suokas et al., 2022). People with severe mental disorders too often have a sedentary lifestyle and do not perform any kind of physical activity (Sampogna et al., 2022a; Sampogna et al., 2022b; Correll et al., 2022; Højlund et al., 2022). Several psychosocial interventions with a specific focus on physical activity and other healthy lifestyle behaviours—such as quit smoking and balanced diet—have been recently developed for people with severe mental disorders (Masa-Font et al., 2015; Speyer et al., 2016; De Rosa et al., 2017; Swift et al., 2021). These interventions showed promising results in terms of reduction of the long-term morbidity and mortality, but a few data are available on their efficacy on adherence to treatments. Indeed, an emerging body of research has linked both the onset and symptoms of various mental disorders to “lifestyle factors”, a term referring to health behaviors such as physical activity, diet, tobacco smoking and sleep and therefore the innovative field of lifestyle psychiatry is nowadays very active and expanding quickly (Firth et al., 2020).

The present research project entitled “LIFESTYLE trial” is a multicentric, randomized controlled study aiming to test the efficacy of an innovative psychosocial intervention on several lifestyle behavioural domains (Sampogna et al., 2018). This paper aims to: 1) assess the level of adherence in a real-world sample of patients with severe mental disorders; 2) evaluate differences in adherence to pharmacological treatments according to patients’ socio-demographic and clinical characteristics; 3) evaluate the impact of an innovative psychosocial intervention on patients’ adherence to treatments.

## Methods

The LIFESTYLE trial was coordinated by the University of Campania “Luigi Vanvitelli” in Naples and carried out in the mental health units of the Universities of Bari, Genova, L’Aquila, Pisa, and Rome-Tor Vergata (Sampogna et al., 2018; Luciano et al., 2022).

The full methodology of the study is available in Sampogna et al. (2018). Patients were included if they met the following criteria: 1) age between 18 and 65 years; 2) diagnosis of schizophrenia, schizoaffective disorder, delusional disorder, other psychotic disorder, major depressive disorder, or bipolar disorder, according to the DSM-5 criteria and confirmed by the Structured Clinical Interview for DSM-5 (SCID-5); 3) ability to provide written informed consent; 4) BMI ≥ 25; 5) in charge at the local mental health unit for at least 3 months before recruitment. Exclusion criteria were: inability to perform moderate physical activity (i.e., walking at least 150 min per week, or 75 min of vigorous activity twice a week, according to the guidelines of the Italian Ministry of Health); pregnancy or breast-feeding; intellectual disability or severe cognitive impairment; hospital admission in the previous 3 months.

The main outcome measure considered for the present analyses is a change of global score at the Morisky Medication Adherence Scale (MMAS) (Morisky et al., 1986), which evaluates the levels of adherence to pharmacological treatments. The MMAS-4 uses a scoring scheme of “Yes” = 0 and “No” = 1. Therefore, the items were summed up to obtain scores ranging from 0 to 4 (e.g., a score of 0 was considered poor; while a score of 4 was considered complete adherence).

Besides the MMAS, all recruited patients were assessed through the following tests: a) the International Physical Activity Questionnaire (IPAQ)—short form (Craig et al., 2003); b) the Food Frequency Questionnaire—short version (Marventano et al., 2016); c) the 24-item Questionnaire on lifestyle behaviours, developed by the Italian National Institute of Health (ISS, 2010); d) the Fagerström Test for Nicotine Dependence (FTND) (Heatherton et al., 1991); e) the Pittsburgh Sleep Quality Index (PSQI) (Buysse et al., 1989); f) the Leeds Dependence Questionnaire (LDQ) (Raistrick et al., 1994); g) the Recovery Style Questionnaire (RSQ) (Drayton et al., 1998); h) the Cumulative Illness Rating Scale (CIRS) (Linn et al., 1968); i) the Manchester Short Assessment of Quality of Life (Priebe et al., 1999); j) the Measurement and Treatment Research to Improve Cognition in Schizophrenia (MATRICS) Consensus Cognitive Battery (MCCB)—brief version (Kern et al., 2008); k) the Internalized Stigma of Mental Illness (ISMI) (Ritsher et al., 2003); l) an *ad hoc* questionnaire on sexual health; m) the Pattern of Care Schedule (PCS)—modified version (Morosini et al., 2000); n) the 24-item Brief Psychiatric Rating Scale (BPRS) (Lukoff et al., 1986); o) the Personal and Social Performance Scale (Morosini et al., 2000).

Information on weight, height, BMI, waist circumference, blood pressure, resting heart rate, HDL, LDL and overall levels of cholesterol, blood glucose, triglycerides, and blood insulin were collected by researchers with an Anthropometric schedule.

All patients have been assessed at the baseline (T0); 2 months post-randomization (T1); 4 months post-randomization (T2); 6 months post-randomization (T3); 12 months post-randomization (T4); and 24 months post-randomization (T5). T1 and T2 assessments include only anthropometric tests. For the scope of the present study, only data collected at baseline and 6 monts post-randomization have been considered.

Patients were randomly allocated to receive the Lifestyle Psychosocial Group Intervention (experimental group) or a Brief Psychoeducational Group Intervention (control group).

The Lifestyle Psychosocial Group Intervention consists of group sessions, delivered every 7–10 days for about 6 months, focused on different lifestyle behaviours, including healthy diet; physical activity; smoking habits; medication adherence; risky behaviours; and regular circadian rhythms. At end of each session a 20-min moderate physical activity is performed by the whole group.

The control Brief Psychoeducational Group Intervention consists of group sessions, delivered every 7 days for about 2 months and focusing on healthy lifestyle; early detection of clinical relapses; effects of pharmacological treatment and management of side effects; stress management techniques; and problem-solving techniques. The interventions were delivered by trained psychiatrists, attending an *ad hoc* brief course on the main characteristics of the interventions. All characteristics of the two interventions are reported in Sampogna et al. (2018).

This study was conducted in accordance with globally accepted standards of good practice, in agreement with the Declaration of Helsinki and with local regulations. The study protocol was formally approved by the Ethics Committee of the Coordinating Center in January 2017 (approval number: 64). Trial registration number is 2015C7374S.

### Statistical analyses

Statistical analyses were conducted according to the “Intention To Treat” principle. Missing data were handled using the Last Observation Carried Forward. Descriptive statistics and frequency tables were used to assess patients’ socio-demographic and clinical characteristics. Chi-square with multiple comparisons and ANOVA with Bonferroni corrections were adopted to detect differences in the levels of adherence to treatments. Bivariate analyses were performed in order to evaluate the association between levels of adherence and severity of clinical symptoms. Descriptive statistics (frequency table, means and standard deviation) were calculated for both experimental and control groups at baseline and at the end of the intervention. Differences in sociodemographic and clinical characteristics among the two groups at baseline and at the end of the intervention were tested using χ^2^ or t-test for independent samples, as appropriate.

Generalized estimating equation (GEE) models were used for evaluating the impact of the experimental intervention on the primary outcome. GEE models allow estimation of population-averaged models in repeated-measures data. Control vs. intervention interaction terms assessed changes between groups over time; Wald tests determined whether joint effects of time-by-group equalled zero. Age and center were included as time-invariant covariates; time-varying covariates included medications, cognitive functioning, age, gender, and diagnosis of mental disorder. GEE models with a normal distribution and identity link were used. Covariate-adjusted results using robust estimates of standard errors are reported. All models were adjusted for diagnosis, pharmacological treatments, duration of illness, and educational level. Pharmacological treatments (i.e., mood stabilizers, tricyclic antidepressants, new-generation antidepressants, first- and second-generation antipsychotics) and psychiatric diagnoses (i.e., depressive disorder, bipolar disorders, psychosis) were included in the regression models as dummy variables.

The level of statistical significance was set at *p* < 0.05 and statistical analyses were performed using the Statistical Package for Social Sciences (SPSS), version 26.0, and STATA, version 15.

## Results

The sample consists of 401 patients, with a mean age of 45.6 years (±11.8), mainly female (57%, N = 227), single (71.4%, N = 287), and unemployed (64.3%, N = 258). The mean duration of illness was 16.3 (±17.8) years, with a median value of 15 years, Inter Quartile Range, INR: 6;23; patients were in charge at the local mental health centre for 5.9 (±6.9) years, with a median value of 3 years (IQR: 1; 9), with a diagnosis of bipolar disorder (43.4%; N = 174), psychotic spectrum disorders (29.6%; N = 120) and major depressive disorder (27.1%; N = 108) (Table 1).

**TABLE 1:** Patients’ socio-demographic and clinical characteristics.

	Global sample (N = 401)	Experimental group (N = 206)	Control Group (N = 195)
Gender, female, % (N)	57 (227)	55.3 (114)	59.0 (115)
Age, M (sd)	45.8 (11.8)	45.9 (11.6)	45.3 (12.1)
Living situation, % (N) Single Married/with partner	71.4 (287)	26.3 (54)	31.3 (61)
	28.6 (115)		
Years of education, M (sd)	11.7 (2.9)	11.8 (2.7)	11.5 (2.9)
Employed, yes, % (N)	35.7 (143)	37.6 (77)	33.8 (66)
Diagnosis, % (N) Bipolar disorder Schizophrenia and other psychotic disorders Major Depression	43.3 (174)	43.2 (89)	43.6 (85)
	29.6 (120)	32.0 (66)	27.2 (53)
	27.1 (108)	24.8 (51)	29.2 (57)
Years in charge to the mental health service, M (sd)	5.9 (6.9)	6.6 (8.1)	7.4 (8.3)
Duration of illness, M (sd)	15.6 (11.3)	16.2 (11.7)	16.4 (22.3)
Number of hospitalizations, M (sd)	2.8 (5.1)	2.1 (4.1)	2.4 (4.3)
Suicide attempts, M (sd)	1.8 (1.6)	1.7 (3.1)	1.8 (3.0)
BPRS, Positive symptoms, M (sd)	5.4 (2.1)	5.5 (3.1)	5.3 (2.1)
BPRS, Negative symptoms, M (sd)	7.7 (3.1)	7.7 (3.0)	7.6 (2.9)
BPRS, Depressive/anxiety symptoms, M (sd)	8.8 (3.1)	8.6 (3.0)	8.7 (3.1)
BPRS, Manic/hostility symptoms, M (sd)	4.7 (1.9)	4.7 (1.9)	4.7 (1.9)
MANSA, Total score, M (sd)	4.1 (1.0)	4.0 (1.1)	4.2 (1.0)
MMAS, total score, M (sd)	1.06 (1.1)	1.1 (1.2)	1.2 (1.1)
B-MCCB, Symbol coding, M (sd)	36.9 (50.3)	34.5 (14.1)	39.4 (70.5)
B-MCCB, Animal naming, M (sd)	20.3 (49.3)	18.2 (51.7)	22.5 (70.4)
B-MCCB Trial making test A, M (sd)	69.1 (127.9)	69.1 (127.9)	69.1 (127.9)
PSP, Total score, M (sd)	65.5 (15.1)	64.5 (14.1)	65.5 (16.2)
Typical Antipsychotics, yes % (N)	22.5 (90)	22.3 (46)	20 (39)
Atypical Antipsychotics, yes % (N)	59 (236)	61.7 (127)	57.4 (112)
First generation antidepressants, yes % (N)	5.7 (23)	6.3 (13)	5.1 (10)
Second generation antidepressants, yes, % (N)	51.5 (205)	45.6 (94)	47.2 (92)
Benzodiazepine, yes % (N)	47.1 (189)	47.1 (97)	46.2 (90)
Mood stabilizers, yes %(N)	65.8 (264)	55.3 (114)	54.4 (103)

The levels of anxiety/depressive symptoms were moderate (8.8 ± 3.1) as well as the level of personal functioning (65.5 ± 15.1 at the PSP scale). All patients were receiving a pharmacological drug treatment; in particular, 59% (N = 236) were treated with a second-generation antipsychotic and 65.8% (N = 264) with a mood stabilizer.

**FIGURE 1:**
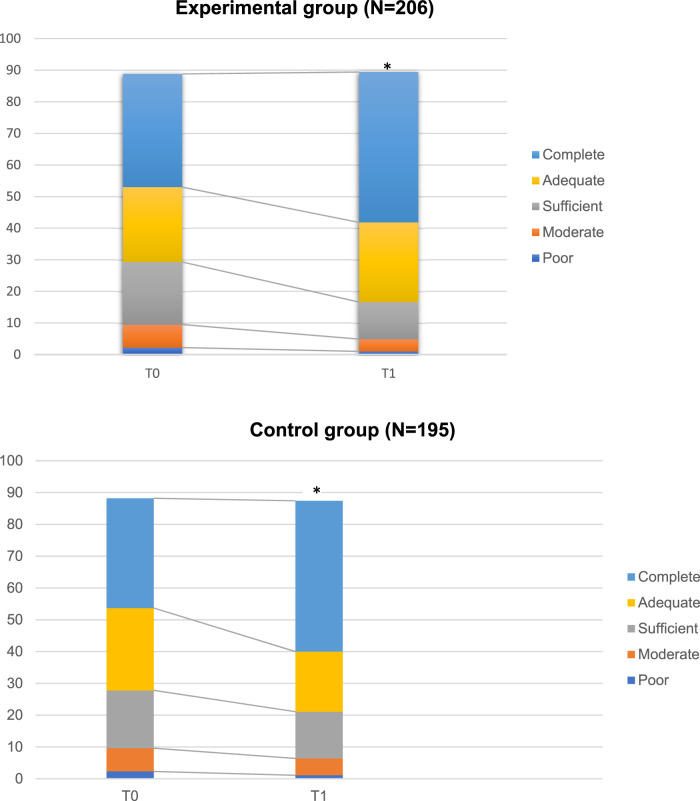
Levels of adherence to pharmacological treatments in the study sample. The weight of the different bars corresponds to the frequency of responses in the different categories of adherence, as evaluated at the Morisky scale. In particular, poor category indicates a condition where all items were scored as “yes”, while “complete adherence”, indicates a condition where all items were scored as “no” *p < 0.005.

39.8% of patients reported a good adherence to the prescribed pharmacological treatments (Figure 1). At bivariate analyses, age, gender, duration of illness and type of the disorder did not influence patients’ adherence to medications. A significant inverse correlation was found between adherence and quality of life (Rho di Person: −0.140, *p* <.005).

The majority of patients were obese (63.1%, N = 253), with a mean BMI of 32.2 (0.2); 53.4% of them were suffering from the metabolic syndrome. All metabolic parameters have been reported in Table 2. Although 29.4% of patients (N = 118) declared to perform physical activity, only 16.1% were found to be very physically active at the IPAQ scale. The most frequently practiced sport activities were walking (52.1%, N = 62), going to the gym (21.8%, N = 26) and playing football (7.5%, N = 9).

**TABLE 2:** Metabolic parameters.

		Baseline				End of intervention			
	Global sample	Experimental		Control		Experimental		Control	
Abd. Circum., M (SD)	108.3 (0.5)	108.64	14.370	109.8	13.65	107.8	12.79	109.5	13.97
**BMI, M (SD)**	**32.2 (0.2)**	**32.2**	**0.36**	**32.9**	**0.41**	**31.60**	**0.46**	**32.83**	**0.67**
Glycemia, M (SD)	94.5 (1.1)	95.32	20.90	95.56	32.30	96.04	25.93	99.13	42.21
Insuline levels, M (SD)	16.76 (0.7)	16.90	19.32	17.78	17.2	16.45	16.63	23.82	30.77
Trygliceric levels, M (SD)	165.6 (5.0)	180.86	159.16	161.0	87.72	172.7	129.9	154.4	81.18
Colesterol levels, M (SD)	190.5 (1.7)	192.7	42.0	186.86	39.62	188.2	36.00	187.3	41.90
LDL levels, M (SD)	121.2 (1.9)	120.98	36,095	117.4	33.68	126.8	101.9	121.4	41.15
HDL levels, M (SD)	46.4 (0.5)	46.16	14.55	45.87	14.74	46.46	14.17	46.11	14.98
Systolic pressure, M (SD)	124.7 (0.5)	125.58	13,638	125.6	13.4	124.2	11.79	124.7	11.93
Dyastolic pressure, M (SD)	80.1 (0.3)	81.14	9,329	80.34	8.60	79.9	7,472	78.8	11.59
Inactive <700 MET	56.7	55.9		58.2		50.6		54.7	
Active 701-2519 MET	27.2	29.1		24.7		29.9		28.4	
Very active > 2520 MET	16.0	15.1		17.1		19.5		16.8	

Bold characters indicate significant differences (*p* < 0.05).

34.5% of patients (N = 138) reported a frequent use of alcohol; 41% of them (N = 163) declared to smoking and 36.3% of them never tried to quit smoking.

There were no significant differences between the experimental (N = 206) and the control (N = 195) groups in any of the considered domains. Further data are available in Supplementary Table S1.

### Longitudinal evaluation of levels of physical activity and adherence to treatments

Hundred and three patients from the experimental group and 95 patients from the control group were re-assessed at 6 months, with an attrition rate of 49.4%. Drop-outs were due to lack of time, reduced interest in the intervention, other personal commitments, and clinical relapses.

At 6 months, the levels of adherence to pharmacological treatment changed from 35.8% at baseline to 47.6% at the end of the intervention (*p* <.005) in the experimental group, while the levels of physical activity did not change between baseline (T0: 6.3%) and 6- month (T1: 9.7%), although a reduction of BMI, weight and other metabolic parameters was found.

The GEE model showed a significant effect of physical activity on adherence to treatments. In particular, at the end of the intervention, patients performing moderate physical activity reported a two-point improvement of adherence to pharmacological treatments (odds ratio [OR]: 1,542; 95% confidence intervals [CI]: 1,157–2,055; *p* < 0.001). Other factors positively influencing adherence were having a diagnosis of major depression (*p* < 0.001), a better cognitive functioning (*p* < 0.001) and quality of life (*p* < 0.05), a shorter duration of illness (*p* < 0.001) and time in contact with the local mental health centre (p < 0.001). Surprisingly, treatment adherence was not influenced by symptom severity and type of pharmacological treatment (Table 3).

**TABLE 3:** GEE model for adherence to treatments.

		OR	95% confidence interval	
	Sign		Lower bound	Upper bound
**Experimental treatment**	**0.008**	**1.035**	**0.870**	**1.232**
**Moderate Physical activity**	**0.003**	**1.542**	**1.157**	**2.055**
Gender, ref. female	0.529	0.958	0.838	1.095
Diagnosis, ref. bipolar		1		
Psychosis spectrum	0.867	0.988	0.859	1.136
**Depression**	**<0.001**	**1.426**	**1.188**	**1.712**
Patient’s age	0.551	0.997	0.987	1.007
brief assessment of cognition	0.039	0.998	0.997	1.000
**Category fluency: animal naming**	**<0.001**	**1.001**	**1.001**	**1.001**
Trail making test	0.691	1.000	0.999	1.002
Personal functioning	0.625	1.000	0.999	1.001
Typical antipsychotic	0.669	0.973	0.860	1.102
Atypical antipsychotic	0.524	0.893	0.629	1.266
Mood stabilizer	0.749	0.968	0.793	1.182
**Tryciclic antidepressant**	**0.036**	**0.703**	**0.506**	**0.978**
II generation antidepressant	0.189	0.920	0.811	1.042
BPRS global score	0.008	1.137	1.033	1.251
Comorbidity index_CIRS	0.005	0.925	0.875	0.977
**Quality of life**	**<0.001**	**0.898**	**0.858**	**0.940**
Sleep disturbances	0.431	1.010	0.986	1.034
**Duration of the illness**	**0.042**	**0.997**	**0.994**	**1.000**
**Time in charge to the mental health service**	**0.045**	**0.999**	**0.998**	**1.000**
**Number of voluntary admissions**	**0.479**	**0.993**	**0.974**	**1.012**
**Number of involuntary admissions**	**0.011**	**0.962**	**0.933**	**0.991**
Intercept	0.020	2.893	1.185	7.064

Bold characters indicate significant variables associated with the outcome measure considered.

## Discussion

Patients’ adherence to treatments is a complex phenomenon posing a significant burden on health professionals, users and carers, and on the healthcare system in general (Atreja et al., 2005).

Lack of adherence is associated with negative consequences on patients’ outcomes, including lack of efficacy of treatments, poor clinical outcome, and worsening of patient health status. This clinical worsening usually requires the subsequent prescription of more drugs, increasing dosages of current drugs, cross-titration of more drugs and other add-on or replacement strategies, which can lead to increased healthcare costs, more frequent consultations, higher rates of emergency services and of hospitalization rates (Semahegn et al., 2020; Gosh et al., 2022; McCutcheon et al., 2022).

Several strategies have been developed in recent years to enhance patient adherence, although the target of “complete adherence” to treatments has not been reached yet (Loots et al., 2021).

In our study on real-world patients suffering from severe mental disorders, 40% of participants reported a good adherence to pharmacological treatments. This finding is slightly lower compared to that reported by the WHO in developed countries, who found that “adherence among patients suffering from chronic diseases averages 50%” (WHO, 2003). However, both our and WHO findings highlight the need to improve medication adherence among patients with chronic physical and mental illnesses (Fernandez-Lazaro et al., 2019; Laranjeira et al., 2023). The lower adherence rates found in people with severe mental disorders compared to those reported by people suffering from other chronic conditions can be due to the presence of specific symptoms, such as cognitive impairment in schizophrenia (El-Missiry et al., 2015; Mendes et al., 2019; Senner et al., 2023); inflated mood in bipolar disorder or hopelessness in major depressive disorder (Chauhan et al., 2021). Indeed, in our sample having a psychiatric diagnosis of schizophrenia and/or bipolar disorder did not influence patients’ adherence to treatments, which is partially in line with findings from Ghosh et al. (2022). This would imply that all psychiatric symptoms have the same weight on adherence rates, and that other causes common to all mental disorders can play a role, such as stigma, prejudices and misconceptions against psychiatric treatments (Kamaradova et al., 2016). Informative campaigns should be carried out at the population level in order to reduce such misconceptions, helping people who take these medications not to feel stigmatized (Corrigan, 2022; Sum et al., 2022).

No significant association was found between illness severity and medication non-adherence. However, lack or poor adherence to medications usually worsens illness severity which, in turn, reduces insight into the illness and has significant adverse clinical outcomes (Wu and Moser, 2018).

Moreover, several socio-demographic variables, including patient’s age and gender, as well as levels of personal functioning and presence of any physical comorbidities did not have any specific impact in modifying the levels of medication adherence. In particular, studies evaluating gender-based difference in medication adherence have highlighted that women are consistently less likely than men to be adherent with their diabetes and cardiovascular medications (Venditti et al., 2023). Some authors argued that this difference may be explained by the fact that women experience more drug side effects than men, while others pointed out that differences in medication adherence are largely due to the type of disorders considered. It should be that the core psychopathological features of different mental disorders play a crucial role in modifying medication adherence, more than socio-demographic features (Semahegn et al., 2020).

At the end of the psychosocial intervention, patients showing a significant improvement in treatments’ adherence also reported increased moderate physical activity. This association can be explained considering the multiple components of our experimental intervention, that include specific sessions dedicated to treatment adherence and to physical activity, with a synergic positive effect of both sessions. Several studies showed that patient’s knowledge about treatments is the strongest predictor of adherence (Jankowska-Polańska et al., 2016; López-Pintor et al., 2021; Kanyongo and Ezugwu, 2023), particularly in patients with severe mental disorders, who can have more difficulties than other patients in understanding the need for taking pharmacological drugs. It can be that the improved adherence found in our sample at the end of the intervention is due to the inclusion of psychoeducational components, motivational interview and cognitive-behavioral techniques (Vieta, 2005; Depp et al., 2008; Okazaki et al., 2023). However, this finding deserves confirmation in long-term studies with larger samples.

The positive association between improved adherence and higher levels of moderate physical activity highlights that physical activity improves global health and functional status. Moreover, it also shows that exercise/physical activity training shall be included in the multilevel personalized treatment for people with severe mental disorders, as already happens in other chronic conditions, such as cardiovascular disease and diabetes mellitus. As recently pointed out by the European Association for Sport and Mental Health (EASMH), the dissemination of sport-based psychosocial interventions for people with severe mental disorders in routine clinical practice is still very low, although considerable evidence is accumulating regarding their efficacy (Sampogna et al., 2022c).

The present study has some limitations, which should be acknowledged. First, the inclusion of patients in a stable phase of the disorder might have biased the results, since they may not be the patients usually seen in routine clinical practice. However, this potential bias has been managed by adopting the GEE model for evaluating the effect of the interventions on the primary outcome (i.e., medication adherence); moreover, all statistical analyses have been controlled for confounding variables, such as type of pharmacological treatment and severity of clinical symptoms. Second, adherence to pharmacological treatments has been evaluated only through a self-reported questionnaire, without other objective measures, which might have led to a potential recall bias. However, introducing more sophisticated biological and clinical evaluations might have hampered the conduction of the study, also because the experimental intervention was developed with the aim to be easily used in routine clinical practice, without a sophisticated training for mental health professionals and high costs. A final limitation is the high drop-out of almost 50%. Reasons for such a high attrition rate vary including the duration of the interventions (which are considered too long by many patients), too structured and manualized approaches (which are considered difficult to follow by many patients), difficulties to travel to the place where the intervention is provided or clinical relapses. In particular, the high attrition should have biased towards those patients more prone to follow recommendations regarding medications as well as practicing physical activity. However, the attrition rate found in our study is similar to that found in other studies on psychosocial interventions. Moreover, the sample size was adequate according to the power analysis, which supports the evidence that the moderate physical activity can improve the levels of adherence.

Thus, future approaches should consider to have a lower total number of sessions, a less structured approach, and the inclusion of online sessions to reduce the need to travel biweekly.

## Conclusion

The poor rate of adherence to treatment reported by patients affected from chronic mental and physical disorders is considered by the WHO an “invisible epidemic”. Poor adherence to treatments is one of the most important—yet modifiable—causes of low efficacy of medications, treatment failure, re-hospitalization, delayed remission and recovery. Therefore, the identification of innovative, multilevel, integrated strategies is essential for overcoming this public health emergency (Kestel, 2022). The promotion of moderate physical activity, which was integrated in our experimental intervention, can represent a valid approach to improve treatment adherence in patients with severe mental disorders. Physical activity exercises, which can be easily implemented in routine clinical practice, are associated with improved outcome. Further studies are needed in larger samples and in acutely severe patients with mental disorders.

## LIFESTYLE working group

Giulia Amatori, Department of Clinical and Experimental Medicine, University of Pisa, Pisa, Italy; Ileana Andriola, Department of Translational Biomedicine and Neuroscience, University of Bari Aldo Moro; Emanuela Bianciardi, Department of Systems Medicine, University of Rome Tor Vergata, Rome, Italy; Laura Capobianco, Section of Psychiatry, Department of Neuroscience, Rehabilitation, Ophthalmology, Genetics, Maternal and Child Health, University of Genoa, Italy; Pierluigi Catapano, Department of Psychiatry, University of Campania “L. VanvitellI”, Naples, Italy; Salvatore Cipolla, Department of Psychiatry, University of Campania “L. VanvitellI”, Naples, Italy; Ivan Cremone, Department of Clinical and Experimental Medicine, University of Pisa, Pisa, Italy; Bianca Della Rocca, Department of Psychiatry, University of Campania “L. VanvitellI”, Naples, Italy; Giorgio Di Lorenzo, Department of Systems Medicine, University of Rome Tor Vergata, Rome, Italy; Ramona Di Stefano, Department of Biotechnological and Applied Clinical Sciences, University of L’Aquila, Italy; Francesca Pacitti, Department of Biotechnological and Applied Clinical Sciences, University of L’Aquila, Italy; Pierluigi Selvaggi, Department of Translational Biomedicine and Neuroscience, University of Bari Aldo Moro; Domenico Zampogna, Section of Psychiatry, Department of Neuroscience, Rehabilitation, Ophthalmology, Genetics, Maternal and Child Health, University of Genoa, Italy.

## Data Availability

The raw data supporting the conclusion of this article will be made available by the authors, without undue reservation.
