# Supplementary material for: Physical activity influences adherence to pharmacological treatments in patients with severe mental disorders: results from the multicentric, randomized controlled LIFESTYLE trial
Source: Front Pharmacol. 2023 Dec 11;14:1285383. doi: 10.3389/fphar.2023.1285383 (PMC10752611; doi:10.3389/fphar.2023.1285383)
Supplement: Supplementary file 1 [file Table1.DOCX]

| **S1. Supplementary table 1** | | | | |
| --- | --- | --- | --- | --- |
|  | Experimental group | | Control group | |
|  | Baseline | End intervention | Baseline | End intervention |
| MANSA | 4.2 (0.1) | 5.3 (1.7) | 3.9 (0.1) | 5.4 (1.4) |
| Adherence to pharmacological treatments | 0.71 (0.09) | 1.5 (0.1) | 1.05 (.07) | 0.78 (0.1) |
| IPAQ - vigorous MET | 206.1 (69.4) | 280.19 (83.3) | 365.12 (87.7) | 164.8 (81.9) |
| IPAQ - moderate MET | 238.9 (55.8) | 505.6 (169.6) | 248.7 (85.4) | 347.4 (116.9) |
| IPAQ – walking MET | 659.1 (74.4) | 922.4 (103.1) | 718.9 (94.7) | 1160.5 (167.5) |
